# Contribution of Thrombospondin-1 and -2 to Lipopolysaccharide-Induced Acute Respiratory Distress Syndrome

**DOI:** 10.1155/2021/8876484

**Published:** 2021-04-16

**Authors:** Qiang Li, Xiaoxiao Fu, Jiang Yuan, Shu Han

**Affiliations:** ^1^The Emergency Department, The Second Affiliated Hospital Zhejiang University School of Medicine (SAHZU), Hangzhou, China; ^2^Institute of Anatomy and Cell Biology, Medical College, Zhejiang University, Hangzhou, China; ^3^Department of Pulmonology, The Children's Hospital, Zhejiang University School of Medicine, National Clinical Research Center for Child Health, Hangzhou, China

## Abstract

Thrombospondin (TSP) proteins have been shown to impact T-cell adhesion, migration, differentiation, and apoptosis. Thrombospondin-1 (TSP-1) is specifically upregulated in several inflammatory diseases and can effectively promote lipopolysaccharide- (LPS-) induced inflammation. In contrast, thrombospondin-2 (TSP-2) has been associated with activation of “anti-inflammatory” T-regulatory cells (Tregs). In this study, we investigated the effects of both TSP-1 and TSP-2 overexpression on macrophage polarization and activation *in vitro* and *in vivo*. We analyzed the effects of TSP-1 and TSP-2 on inflammation, vascular endothelial permeability, edema, ultrastructural morphology, and apoptosis in lung tissues of an ARDS mouse model and cultured macrophages. Our results demonstrated that TSP-2 overexpression effectively attenuated LPS-induced ARDS *in vivo* and promoted M2 macrophage phenotype polarization *in vitro*. Furthermore, TSP-2 played a role in regulating pulmonary vascular barrier leakage by activating the PI3K/Akt pathway. Overall, our findings indicate that TSP-2 can modulate inflammation and could therefore be a potential therapeutic target against LPS-induced ARDS.

## 1. Introduction

Acute respiratory distress syndrome (ARDS) is a diffuse alveolar injury caused by lung infection or systemic inflammation/sepsis in multiple patients. Lung inflammation can disrupt alveolar endothelial and epithelial barriers and in turn enhance the influx of multiple cell types, including neutrophils, macrophages, and other inflammatory cells that subsequently release various inflammatory and cytotoxic mediators [1]. Earlier studies reported that macrophages protect against ARDS by modulating inflammation and initiating lung repair [2, 3]. However, it is important to highlight that the role of macrophages in regulating inflammation is dependent on the relative abundance of their specific M1 and M2 phenotypes. M1-polarized macrophages are typically generated in response to microbial products or interferon- (IFN-) *γ*, and can efficiently produce proinflammatory cytokines, including interleukin- (IL-) 6, IL-12, and tumor necrosis factor- (TNF-) *α*. These cytokines induce a polarized type I immune response that inhibits cell proliferation and induces tissue damage. In contrast, M2-polarized macrophages induce anti-inflammatory cytokines and promote tissue remodeling and wound healing [4].

Thrombospondins (TSPs) are nonstructural glycoproteins belonging to a family of matricellular proteins involved in regulating cell-matrix interactions. TSPs have been shown to influence T-cell adhesion, migration, differentiation, and apoptosis [5]. Among the various TSPs, thrombospondin-1 (TSP-1), which is upregulated in several inflammatory diseases [6], can effectively augment lipopolysaccharide- (LPS-) induced inflammation in THP-1 cells. Interestingly, TSP-1 ablation by siRNA has been observed to reduce IL-6, IL-1*β*, and TNF-*α* secretion in THP-1 cells, while its overexpression upregulated these cytokines by regulating the NF-*κ*B signaling pathway [7]. The CD47 protein has been shown to be involved in TSP-1-mediated regulation of IL-1*β* [8]. Thus, TSP-1 and CD47 could be potential targets for limiting the proinflammatory effects of LPS. In contrast, thrombospondin-2 (TSP-2) has been implicated in endogenous regulation of angiogenesis and autoimmune inflammation and appears to be part of the protective mechanism for preventing inflammation [9]. TSP-2 has also been shown to activate anti-inflammatory T-regulatory cells (Tregs) [5]. Importantly, previous studies have also revealed that TSP-2 ablation significantly decreased the expression of phosphorylated- (p-) Akt and p-phosphatidyl inositol-3 kinase (PI3K), thereby indicating its role in PI3K/Akt pathway activation [10].

Despite information about the pro- and anti-inflammatory roles of TSP-1 and TSP-2, their relationship with M1/M2 macrophages is still unclear. Moreover, their potential role in the pathological process of ARDS requires further investigation. Therefore, in our current study, we investigated the effects of both TSP-1 and TSP-2 overexpression on macrophages *in vitro* and *in vivo* using a well-established LPS-induced ARDS mouse model. We also investigated the role of TSP-1 and TSP-2 on *in vitro* macrophage polarization and their potential to regulate the PI3K/Akt signaling pathway.

## 2. Materials and Methods

### 2.1. *In Vitro* Experiments

#### 2.1.1. Cell Culture, Transfection, and *In Vitro* LPS Stimulation

The MH-S murine alveolar macrophage cell line purchased from American Type Culture Collection (Manassas, VA) was initially cultured in RPMI 1640 medium containing 10% heat-inactivated fetal calf serum (FCS) (Hyclone Laboratories, Logan, UT). Later, these cells were plated on 6-well tissue culture plates at a concentration of 5 × 10^5^ cells/ml in 5% CO_2_ at 37°C. Cell monolayers were washed with Hank's buffered saline solution (HBSS) and replenished with 10% FCS-RPMI 1640 media without antibiotics for subsequent transfection, as described previously [11]. Full-length human TSP-1 and TSP-2 cDNA constructs subcloned into the pEGF expression vector were transfected into the MH-S cells. The empty vector was transfected into MH-S cells as a control. These *pEGFP-TSP1* and *pEGFP-TSP2* cDNAs were mixed with Lipofectamine PlusTM Reagent (Life Technologies) in serum and antibiotic-free media for 15 min at room temperature and then added to the plated cells. Forty-eight hours later, visible green fluorescence from the pEGF expression vector was detected in the transfected cells using fluorescence microscopy, and TSP-1 or TSP-2 protein expression was detected using Western blot analysis. For *in vitro* stimulation, MH-S cells overexpressing TSP-1 and TSP-2 were first serum starved for 24 h and then treated with LPS (L, 10 *μ*g/ml) for 30 min. MH-S cells expressing empty vector were treated with LPS as “vehicle control” or with culture medium as “normal control.”

#### 2.1.2. Immunofluorescence Staining

After fixing cultured cells with 4% paraformaldehyde for 30 min, the cells were washed with phosphate-buffered saline (PBS) and later incubated with primary antibodies against polyclonal rabbit anti-TSP-1 and TSP-2 (1 : 100; Chemicon, Euromedex, Souffelweyersheim, France), anti-CD38 (1 : 100; Santa Cruz Biotechnology), anti-Akt and p-Akt (1 : 500; R&D Systems, Minneapolis, MN, USA), anti-arginase-1 (Arg-1, 1 : 500; R&D Systems, Minneapolis, MN, USA), anti-formyl peptide receptor 2 (Fpr-2, 1 : 500; R&D Systems, Minneapolis, MN, USA), anti-active caspase-3 (1 : 100; Santa Cruz Biotechnology, Santa Cruz, CA), and anti-early growth response protein 2 (EGR2, 1 : 200; Cayman Chemical, Ann Arbor, MI, USA), as described previously [12]. Omitting primary antibody controls was used to confirm the specificity of staining signals. Five areas from each slide were randomly selected for image acquisition under 200x magnification. The red immunoreactive signal indicated expression of each specific protein, while the green signal indicated TSP-1 and TSP-2 expression; the Hoechst 33342 blue signal indicated nuclei staining.

#### 2.1.3. Enzyme-Linked Immunosorbent Assay (ELISA)

The cultured supernatants from each group of cells were used to assess TNF-*α*, IL-6, and IL-1*β* concentrations using an ELISA kit according to the manufacturer's instructions (BioLegend, Inc., USA).

### 2.2. *In Vivo* Experiments

#### 2.2.1. Experimental Design

A total of 320 C57/BL6 mice were randomly divided into four groups. Three groups (100 mice per group) were injected *via* the tail vein with cDNA of TSP-1, TSP-2, or vehicle (empty vector), while the fourth group (20 mice) served as the normal group. The Entranster™-*in vivo* kit (Engreen Biosystem Ltd., Beijing, China) was used to transfect macrophages in the host *in vivo* to overexpress TSP-1 and TSP-2. After mixing the cDNA of TSP-1, TSP-2, or the empty vector with the Entranster kit solutions, the mixture was injected directly into the tail veins of the mice. ARDS was induced by intravenous injection of LPS (4 mg/kg) (L4391, Sigma-Aldrich) under anesthesia with intraperitoneal sodium pentobarbital (30 mg/kg) in the TSP-1, TSP-2, and vehicle-transfected mice. To specifically observe the effects of the PI3K/Akt pathway on lung inflammation, we injected TSP-1- and TSP-2-transfected mice with LY294002 (0.3 mg/kg) *via* the tail vein. All animal experiments were performed according to the protocols and policies described in the Care and Use of Laboratory Animals issued by the National Institutes of Health and approved by the animal ethics committee of Zhejiang University.

#### 2.2.2. Histopathological Examination and Immunohistochemical (IHC) Staining

Five mice from each of the TSP-1 cDNA, TSP-2 cDNA, and vehicle groups were sacrificed after 4, 12, 24, and 72 h of LPS injection. To perform histopathological examination, the animals were anaesthetized using ketamine-xylazine (120 mg/kg ketamine hydrochloride (5%) and 1 mg/kg xylazine (2%)), and the trachea of each mouse was cannulated with an indwelling needle and later perfused with saline. *In situ* fixation was achieved by instillation of 4% paraformaldehyde in PBS. Subsequently, half of the lungs were incised and fixed in 4% paraformaldehyde for 4 h followed by equilibrium with 30% sucrose solution in PBS, while the remaining half was fixed in 10% glutaraldehyde solution for transmission electron microscopy imaging. Frozen sections (20 *μ*m thick) were prepared using a Leica cryostat and mounted on glass slides coated with 0.02% poly-L-lysine for histological analysis and immunofluorescent staining.

Lung histology of hematoxylin and eosin- (H&E-) stained slides was assessed under a light microscope by an experienced pathologist blinded to the experimental conditions. The degree of lung injury was assessed by two independent pathologists and scored as follows: 0 represented no injury; 1 represented subpleural edema/fibrin and hemorrhage; 2 represented interlobular edema/fibrin and hemorrhage; 3 represented alveolar edema/fibrin and hemorrhage; 4 represented congestion of alveolar septa; and 5 represented hyaline membrane changes of the alveolar septa. The overall score from each mouse was averaged within a group to obtain an average score, and comparisons were then made between different groups [13, 14].

IHC staining of the frozen lung sections was performed using the following primary antibodies: rabbit anti-TSP-1 and TSP-2 (1 : 500, Chemicon, Euromedex, Souffelweyersheim, France), anti-Arg1 (1 : 500; Cayman Chemical, Ann Arbor, MI, USA), anti-EGR2 (1 : 500; Cayman Chemical, Ann Arbor, MI, USA), anti-p85 (1 : 200; Abcam, Cambridge, MA, USA), anti-Akt and p-Akt (1 : 500; R&D Systems, Minneapolis, MN, USA), anti-CD38 (1 : 100; Santa Cruz Biotechnology), anti-Fpr-2 (1 : 500; R&D Systems, Minneapolis, MN, USA), anti-CD68 (1 : 100; Santa Cruz Biotechnology), and anti-active caspase-3 (1 : 500; Cayman Chemical), as described previously [12].

#### 2.2.3. Bronchoalveolar Lavage Fluid (BALF) Preparation and Cell Counting

After 4, 12, 24, and 72 h of LPS stimulation (*n* = 3 per group at each time point), the lungs were gavaged three times with a total volume of 1.5 ml cold PBS. Following centrifugation of the gavage fluid at 700 g for 10 min at 4°C, the cell pellets were resuspended in 1 ml of fresh PBS. Cell counting was performed using a hemocytometer in a double-blind manner, while differential cell counting was done using Wright-Giemsa staining (KeyGen Biotech, Nanjing, China).

#### 2.2.4. Assessment of Cytokines in Peripheral Blood

The concentrations of TNF-*α*, IL-1*β*, and IL-10 were measured in the peripheral blood collected from mice after 4, 12, 24, and 72 h (*n* = 5 per group at each time point) of LPS stimulation using ELISA kits (Abcam, Cambridge, UK). The optical density (OD) at 450 nm was used to assess the concentrations of each cytokine using GraphPad Prism 4.0 (GraphPad Software, Inc., San Diego, CA, USA).

#### 2.2.5. Assessment of Wet-to-Dry Lung Weight Ratio (*W*/*D* Ratio)

Lungs from each mouse were immediately dissected after sacrifice, and wet weight (*W*) was recorded. Subsequently, the lung tissues were dried in an 80°C incubator for 48 h, and later, dry weight (*D*) was recorded. The *W*/*D* ratio was calculated to assess lung edema [11].

#### 2.2.6. Evans Blue Extravasation Method to Assess Pulmonary Vascular Permeability

Vascular permeability in each group was assessed using a modified Evans blue extravasation method. Briefly, mice (*n* = 5 from each group) were anesthetized with sodium pentobarbital (60 mg/kg, i.p.), and Evans blue dye (2% in 0.9% normal saline, 4 ml/kg) was infused at 37°C via the right femoral vein within a 5 min time window. Two hours later, the mice were perfused with 300 ml of normal saline to flush out any remaining dye in the blood vessels. Pulmonary vascular permeability was then evaluated in the lung tissues. To assess pulmonary vascular permeability, half of the lung tissue was removed and mechanically homogenized in 750 *μ*l of *N*,*N*-dimethylformamide (DMF; Sigma-Aldrich, St. Louis, MO). The suspended material was maintained at room temperature in the dark for 72 h and later centrifuged at 10,000 × *g* for 25 min. The supernatant was subsequently analyzed using a spectrophotometer (Molecular Devices OptiMax, USA) at 610 nm OD. The overall dye concentrations were expressed as *μ*g/g of tissue weight and calculated from a standard curve prepared using known concentrations of the dye [15]. The remaining left lung tissue was sectioned (20 *μ*m thick) for digital imaging.

#### 2.2.7. Electron Microscopy (EM)

Tissue sections were fixed in 10% glutaraldehyde and analyzed using a transmission electron microscope, as described previously [12].

#### 2.2.8. Western Blot Analysis

The protein lysates from mouse lung tissue sections were prepared as described previously [15]. As a negative control, the membrane was probed in the absence of a primary antibody.

#### 2.2.9. Statistical Analyses

All quantified results are presented as mean ± standard deviation (SD). To compare groups, one-way analysis of variance (ANOVA) and the Student-Newman-Keuls (SNK) pairwise tests were used. A *P* value of < 0.05 represented statistical significance. All statistical analyses were conducted using SPSS 11.5 software package, and graphs were generated using GraphPad Prism Version 4.0 software (GraphPad Software, Inc. CA, USA).

## 3. Results

### 3.1. *In Vitro* Overexpression of TSP-2 Promotes M2 and TSP-1 Promotes M1 Macrophage Polarization

**Figure 1:**
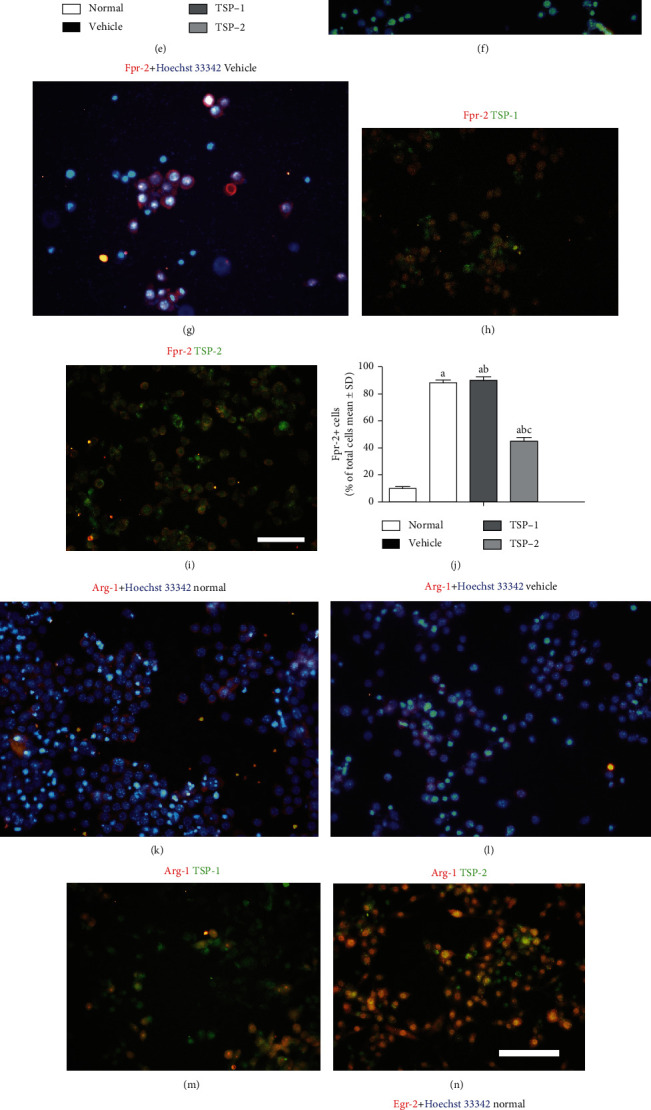
TSP-1 and TSP-2 overexpression affects macrophage polarization *in vitro*. MH-S murine alveolar macrophage cells were either untreated (normal and nontreatment) or treated with LPS (1 *μ*g/ml) for 24 h following either empty vector (vehicle group), TSP-1 (TSP-1 group), or TSP-2 (TSP-2 group) overexpression. Cells were harvested and used for immunostaining of M1 (CD38 and Fpr-2) and M2 (Agr-1 and Egr-2) macrophage markers. (a–d) CD38 staining; (f–i) Fpr-2 staining; (k–n) Agr-1 staining; (p–s) Egr-2 staining in normal, vehicle, and TSP-1- and TSP-2-overexpressing cells, respectively. Red staining represents expression of specific markers, Hoechst 33342 blue staining represents the nuclei, and green staining represents GFP-labeled TSP-1 and TSP-2 overexpression. The yellow color depicts overlay image of specific markers and TSP expression in the same cell. Positively stained cells for specific markers in each group were counted and are shown in a bar graph in (e, j, o, and t). The percentage of labeled cells/total cell numbers is shown as mean ± SD. *P* value of < 0.05 represents significant difference. ^a^*P* < 0.05 compared with normal control; ^b^*P* < 0.05 compared with vehicle group; ^c^*P* < 0.05 compared with TSP-1 transfected group (*n* = 5 per group).

After LPS treatment (vehicle) of MH-S cells transfected with empty vector, the expression of M1 phenotype markers, CD38 and Fpr-2, increased compared to cells that were not treated (normal) (Figures 1(a), 1(b), 1(f), and 1(g)). In addition, LPS treatment of TSP-1-overexpressing MH-S cells did not alter CD38 and Fpr-2 expression (Figures 1(c) and 1(h)), but TSP-2 overexpression reduced CD38 and Fpr-2 expression compared to vehicle control (Figures 1(d) and 1(i)). The overall comparison of M1 markers, CD38 and Fpr-2, is shown in Figures 1(e) and 1(j).

Similarly, expression of M2 phenotype markers, Arg-1 and Egr-2, was reduced in LPS-treated MH-S cells compared to cells that were not treated (Figures 1(k), 1(l), 1(p), and 1(q)). However, LPS treatment of TSP-1-overexpressing MH-S cells did not significantly change Arg-1 and Fpr-2 expression compared to vehicle-treated cells (Figures 1(m) and 1(r)). Interestingly, LPS-treated TSP-2-overexpressing MH-S cells showed increased expression of Arg-1 and Egr-2 (Figures 1(n) and 1(s)). The overall comparison of M2 phenotype markers, Arg-1 and Egr-2, is shown in Figures 1(o) and 1(t).

### 3.2. TSP-2 Overexpression Attenuates LPS-Induced Apoptosis and Promotes PI3K Signaling *In Vitro*

**Figure 2:**
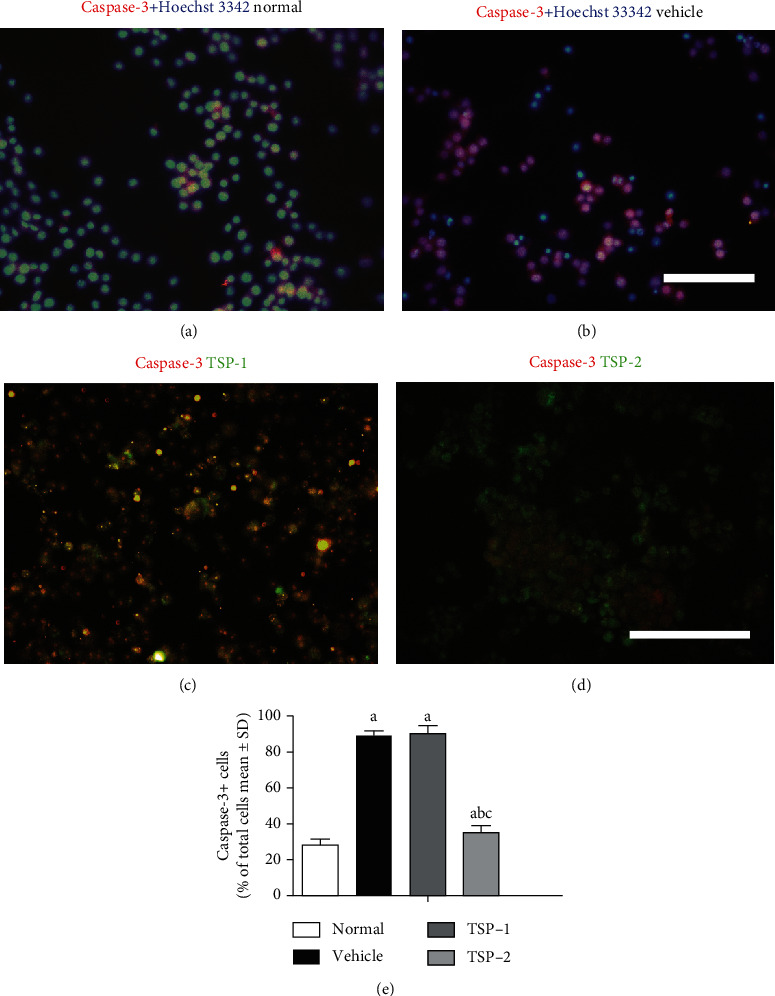
Effect of TSP-2 overexpression on LPS-induced apoptosis. MH-S murine alveolar macrophage cells were either untreated (normal and nontreatment) or treated with LPS (1 *μ*g/ml) for 24 h following either empty vector (vehicle group), TSP-1 (TSP-1 group), or TSP-2 (TSP-2 group) overexpression. Cells were stained with caspase-3 (red) antibody to assess apoptosis (a–d), respectively. Hoechst 33342 dye (blue) was used to stain the nucleus. Green stain represents TSP-1 and TSP-2 overexpression. The yellow color depicts overlay image of caspase-3 and TSP expression in the same cell. (e) Bar graph representing overall quantitation and parallel comparison of caspase-3 staining among the four groups. The percentage of labeled cells/total cell numbers are shown as mean ± SD. *P* value of < 0.05 represents significant difference. ^a^*P* < 0.05 compared with normal control; ^b^*P* < 0.05 compared with the vehicle group; ^c^*P* < 0.05 compared with the TSP-1 transfected group (*n* = 5 per group).

Next, we analyzed the effects of TSP-1 and TSP-2 overexpression on apoptosis in MH-S cells after LPS induction by analyzing caspase-3 marker staining. Our data showed that LPS treatment itself induced the percentage of caspase-3-positive cells compared to no treatment (normal MH-S cells). However, TSP-1-overexpressing MH-S cells after LPS treatment showed a similar percentage of caspase-3-positive staining, but TSP-2-overexpressing MH-S cells showed a significantly reduced percentage of caspase-3-positive cells compared to LPS-treated MH-S cells expressing empty vector (Figures 2(a)–2(d)). Caspase-3 staining in the four groups of cells is shown in Figure 2(e).

**Figure 3:**
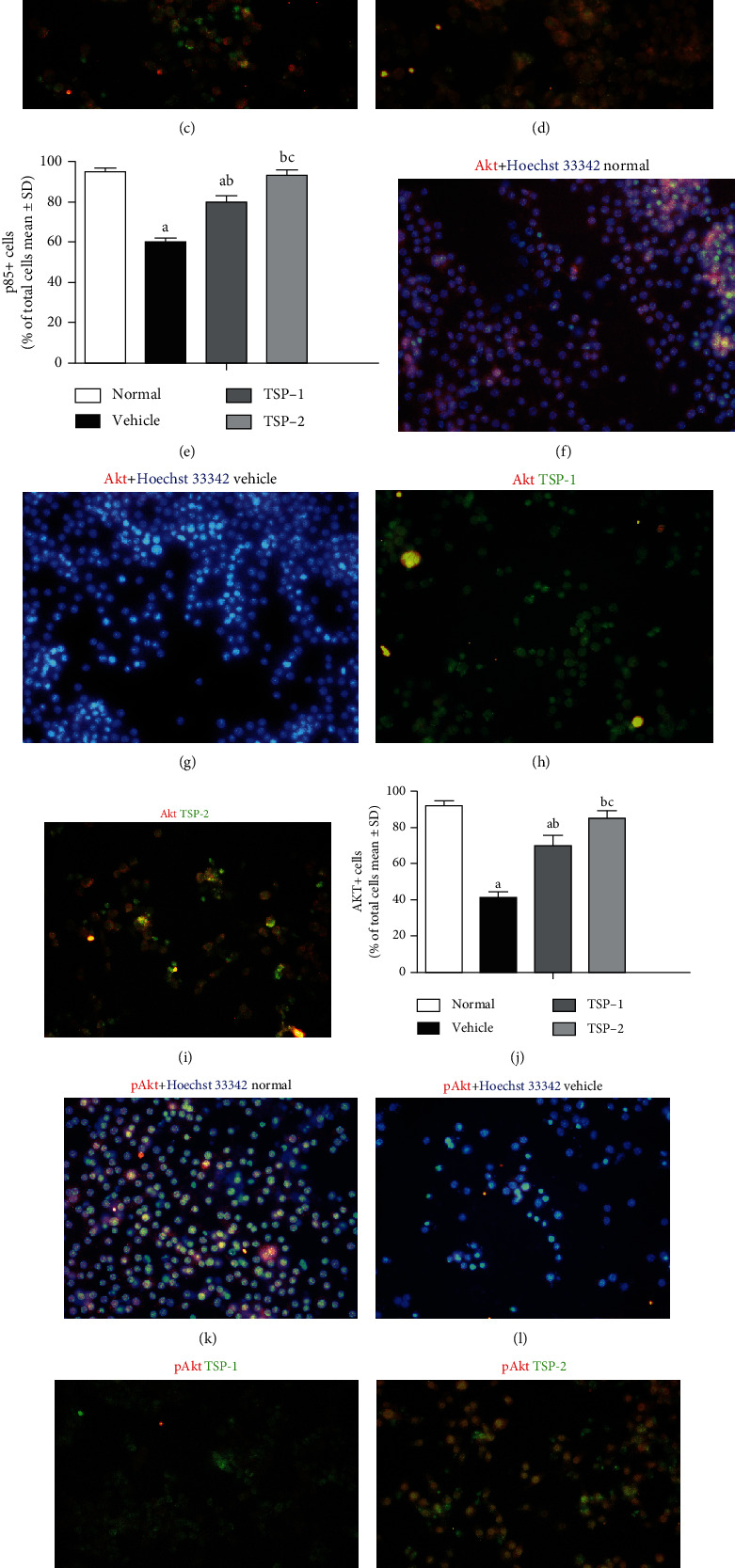
Impact of TSP-2 overexpression on PI3K signaling *in vitro*. MH-S murine alveolar macrophage cells were either untreated (normal and nontreatment) or treated with LPS (1 *μ*g/ml) for 24 h following either empty vector (vehicle group), TSP-1 (TSP-1 group), or TSP-2 (TSP-2 group) overexpression. MH-S cells were stained with p85 (a–d), total Akt (f–i), and p-Akt (k–n) antibodies to assess the impact on PI3K signaling. Red color is antibody-specific staining, blue color is Hoechst 33342 nuclei staining, and green color is GFP-labeled TSP-1- and TSP-2-overexpressing cells. Yellow color shows colocalization of TSP-1 or TSP-2 with specific proteins. (e, j, and o) Bar graphs showing parallel comparisons of antibody staining quantitation among the four groups. The percentage of labeled cells/total cell numbers is shown as mean ± SD. *P* value of < 0.05 represents significant difference. ^a^*P* < 0.05 compared with normal control; ^b^*P* < 0.05 compared with the vehicle group; ^c^*P* < 0.05 compared with the TSP-1 transfected group (*n* = 5 per group).

In addition, we analyzed the effects of TSP-1 and TSP-2 overexpression on the PI3K signaling pathway in LPS-treated MH-S cells. Specifically, we assessed expression of p85, p-Akt, and total Akt using immunostaining. As shown in Figure 3, LPS treatment alone reduced p85 signal intensity (Figure 3(b)), while TSP-1 and TSP-2 overexpression increased the p85 signal (Figures 3(c) and 3(d)). Quantification of p85 staining in the different conditions is shown in Figure 3(e). Similarly, LPS treatment reduced the levels of total Akt, but TSP-1 and TSP-2 overexpression restored Akt levels (Figures 3(f)–3(j)). Moreover, p-Akt expression decreased after LPS treatment (Figure 3(l)), but only TSP-2 overexpression restored p-Akt expression (Figure 3(n)), while TSP-1 overexpression had no effect (Figure 3(m)). The bar graph in Figure 3(o) shows the overall signal intensity of p-Akt among the four groups.

### 3.3. TSP-1 and TSP-2 Overexpression Differentially Affects Cytokine Secretion in the ARDS Mouse Model

**Figure 4:**
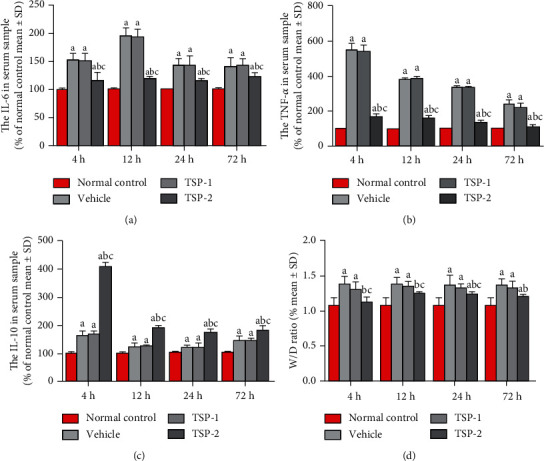
ELISA analyses of inflammatory cytokines in mouse serum and assessment of lung *W*/*D* ratios. (a–c) Bar graphs representing inflammatory cytokines IL-6 (a), TNF-*α* (b), and IL-10 (c) in serum from the normal, vehicle, TSP-1, and TSP-2 groups, using ELISA. (d) Comparison of lung *W*/*D* ratio among the four groups. (a–c) The percentage of expression level of each group/normal control is shown as mean ± SD. (d) The lung *W*/*D* ratio of each group is shown as mean ± SD. *P* value of < 0.05 represents significant difference. ^a^*P* < 0.05 compared with normal control; ^b^*P* < 0.05 compared with the vehicle group; ^c^*P* < 0.05 compared with the TSP-1 transfected group (*n* = 5 per group).

Macrophages from mice injected with TSP-1-overexpressing cells showed significant upregulation of TSP-1, as expected. The bar graphs in Supplementary Figure 1B present the parallel comparison of TSP-1 expression among mice in the different groups. Supplementary Figures 1C and 1D show the upregulation of TSP-2 only in mice injected with TSP-2-overexpressing cells. We next analyzed the serum cytokine levels in the four groups (Figures 4(a)–4(c)). IL-6, TNF-*α*, and IL-10 were induced in mice that were injected with TSP-1 similar to LPS treatment alone. However, TSP-2 overexpression significantly suppressed LPS-induced secretion of inflammatory cytokines IL-6 and TNF-*α*, but upregulated secretion of anti-inflammatory factor IL-10.

### 3.4. TSP-2 Overexpression Inhibits Pulmonary Vascular Permeability and Edema in ARDS Mice

**Figure 5:**
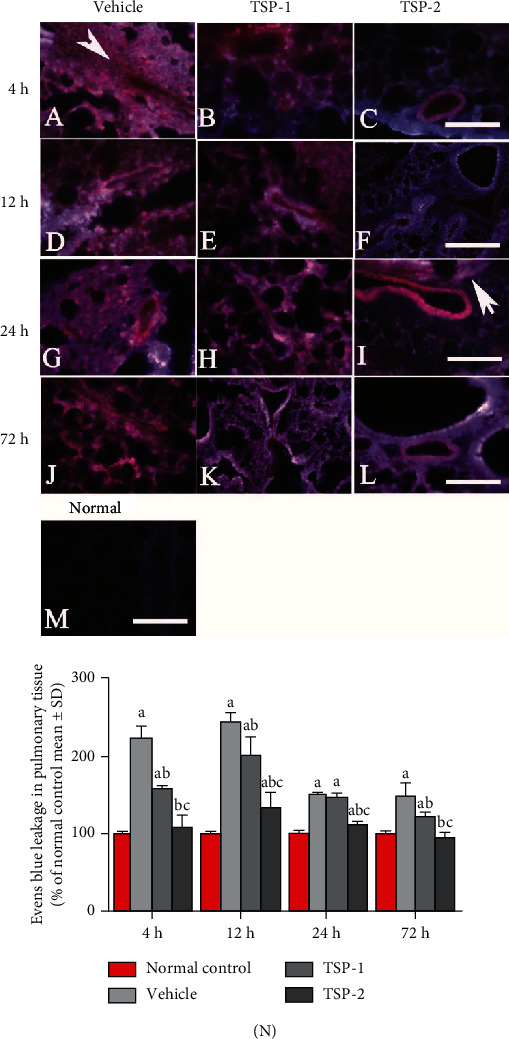
The impact of TSP-2 on pulmonary vascular permeability in the ARDS mouse model. (a–l) Evans blue (EB) staining is shown as an index of pulmonary vascular permeability in lung sections collected from vehicle, TSP-1, and TSP-2-overexpressing mice. (m) Normal mice. (n) Percentage of EB leakage in pulmonary tissue compared to normal control. EB staining was visualized using red laser excitation at 405 nm OD. Blue color represents no EB leakage into the pulmonary tissue, red color depicts high EB leakage due to enhanced vascular permeability, and pink color shows medium vascular permeability. The arrows point to the site of EB leakage. Scale bar = 100 *μ*m and *n* = 5. (n) Evans blue leakage in pulmonary tissue (*μ*g/g) is shown as mean ± SD. *P* value of < 0.05 represents significant difference. ^a^*P* < 0.05 compared with normal control; ^b^*P* < 0.05 compared with the vehicle group; ^c^*P* < 0.05 compared with the TSP-1 transfected group (*n* = 4 per group).

We next assessed the effects of TSP proteins on pulmonary vascular permeability using Evan's blue extravasation assay in the ARDS mouse model. We observed that TSP-2 overexpression significantly reduced pulmonary vascular permeability, as evident from reduced staining (red color) due to poor leakage (Figures 5(c), 5(f), 5(i), and 5(l)) compared to LPS-treated mice transfected with empty vector, which had higher staining (red color) due to increased pulmonary vessel permeability at all time points (Figures 5(a), 5(d), 5(g), and 5(j)). However, TSP-1 overexpression resulted in higher pulmonary vascular permeability compared to the TSP-2 overexpression group.  Figure 5(m) shows the basal level of staining in normal mice without any LPS treatment. The bar graphs in Figure 5(n) represent the normalized dye leakage in the different experimental conditions. Similarly, we assessed the effects of TSP overexpression on pulmonary edema by measuring the *W*/*D* ratio of lungs in the ARDS mice. As shown in Figure 4(d), TSP-2 overexpression reduced the *W*/*D* ratio, while TSP-1 overexpression had similar effects compared to LPS treatment alone.

### 3.5. TSP-2 Overexpression Inhibits Pulmonary Inflammation and Lung Injury in ARDS Mice

**Figure 6:**
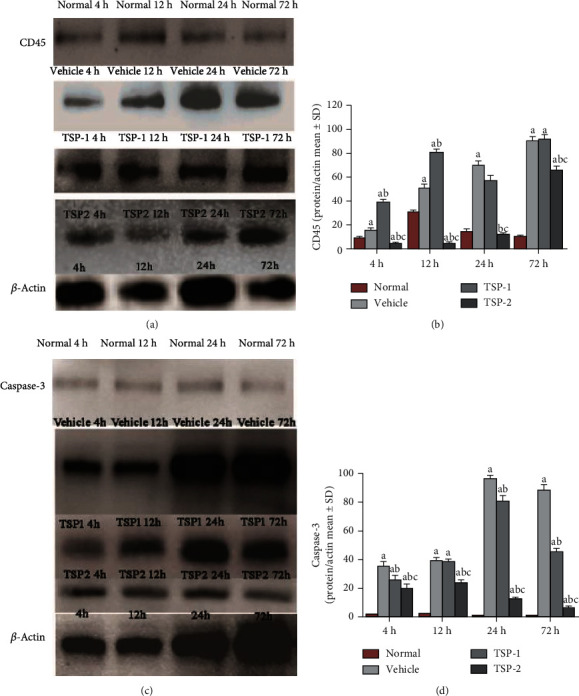
CD45 and caspase-3 protein expression in lung tissue lysates. Mouse protein lysates from normal mice or mice treated with LPS for 4, 12, 24, and 72 h and overexpressing either empty vector (vehicle group), TSP-1 (TSP-1 group), or TSP-2 (TSP-2 group) were analyzed for CD45 (a) and caspase-3 (c) protein expression using Western blot analysis. *β*-Actin expression served as the loading control. (b, d) Bar graphs representing CD45 and caspase-3 expression after normalizing to *β*-actin expression among four different groups. The percentage of detected protein/actin is shown as mean ± SD. *P* value of < 0.05 depicts significant difference. ^a^*P* < 0.05 compared with normal control; ^b^*P* < 0.05 compared with vehicle group; ^c^*P* < 0.05 compared with TSP-1 transfected group (*n* = 4 per group).

To understand the role of TSP proteins in pulmonary inflammation, we analyzed the expression of CD45, a specific marker of leukocytes in lung tissue, using Western blot analysis. CD45 expression increased in lung tissue sections after LPS stimulation in the vector and TSP-1-overexpressing mice, but significantly decreased in the TSP-2-overexpressing mice, as shown in Figures 6(a) and 6(b).

We also analyzed the accumulation of inflammatory cells in the BALF of mice from the four different groups using Wright-Giemsa staining. We observed neutrophil accumulation after 4, 12, 24, and 72 h of LPS treatment in mice expressing empty vector. A similar neutrophil accumulation profile was observed in the BALF collected from TSP-1-overexpressing mice, but the BALF from TSP-2-overexpressing mice displayed a significantly reduced number of neutrophils (Supplementary Fig 2A–N).

**Figure 7:**
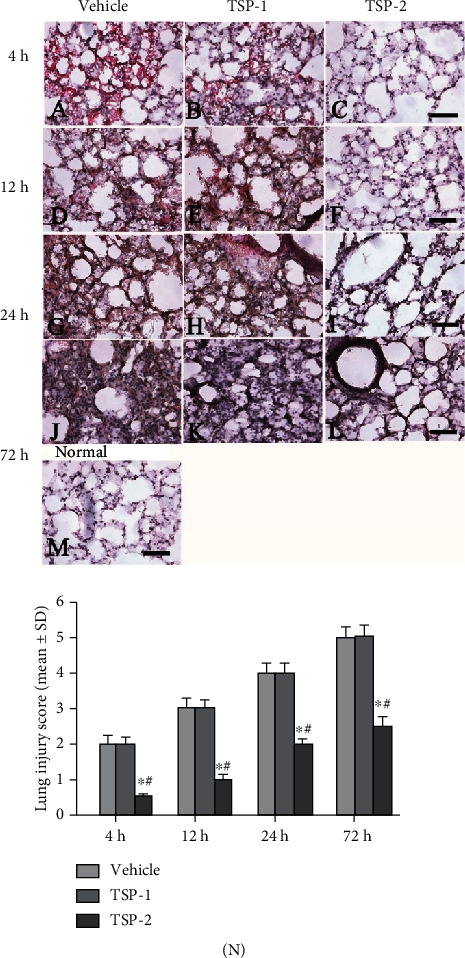
Effect of TSP-2 overexpression on pulmonary morphology in the ARDS mouse model. Lung tissue sections from mice treated with LPS for 4, 12, 24, and 72 h and overexpressing either empty vector (vehicle group), TSP-1 (TSP-1 group), or TSP-2 (TSP-2 group) were analyzed for pulmonary morphology using H&E staining (a–l). (m) Normal control: the normal morphology of blood vessels, bronchi, and interstitium. Scale bar = 100 *μ*m. (n) Bar graph of lung injury scores from three different groups, assessed by two independent pathologists blinded to the experimental conditions. Scoring was based on the following: 0 = no injury; 1 = edema/fibrin, hemorrhage (subpleural); 2 = edema/fibrin, hemorrhage (interlobular); 3 = edema/fibrin, hemorrhage (alveolar); 4 = congestion of alveolar septa; 5 = hyaline membrane changes of alveolar septa. The lung injury score of each group is shown as mean ± SD. *P* value of < 0.05 represents significant difference. ^a^*P* < 0.05 compared with normal control; ^b^*P* < 0.05 compared with the vehicle group; ^c^*P* < 0.05 compared with the TSP-1 transfected group (*n* = 5 per group).

Next, we investigated pulmonary morphology in ARDS mice from four different groups using H&E staining of lung tissues. We observed alveolar septa thickening and inflammatory cell infiltration after 4 h of LPS treatment in the empty vector and TSP-1-expressing mice (Figures 7(a) and 7(b)), and more severe alveolar hemorrhage and additional thickening were evident at later time points (12, 24, and 72 h) (Figures 7(d), 7(e), 7(g), 7(h), 7(j), and 7(l)). In contrast, TSP-2-overexpressing mice displayed reduced alveolar hemorrhage and septa thickening, as well as less inflammatory cell infiltration at all time points after LPS treatment (Figures 7(c), 7(f), 7(i), and 7(l)). Lung tissue injury scores from mice are shown in Figure 7(n). The effects of TSP-2 on pulmonary histology were consistent with the observed decrease in lung inflammation.

**Figure 8:**
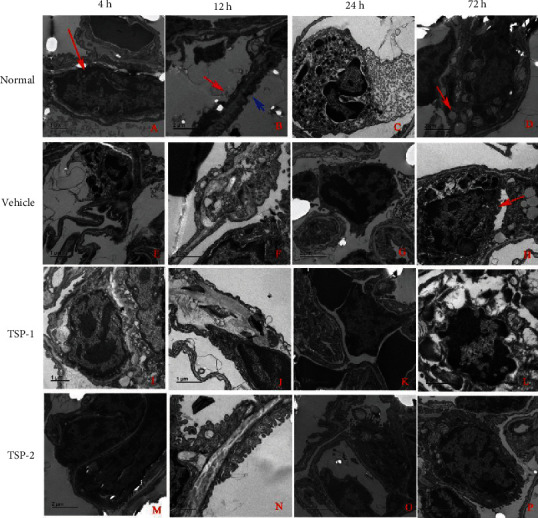
Ultrastructural morphology after TSP-2 overexpression in the ARDS mouse model. Mouse tissue sections from normal mice or mice treated with LPS for 4, 12, 24, and 72 h and overexpressing either empty vector (vehicle group), TSP-1 (TSP-1 group), or TSP-2 (TSP-2 group) were analyzed for ultrastructural morphology using electron microscopy. (a–d) Ultrastructure of pulmonary capillaries under normal conditions. Arrow in (a) shows normal blood vessel epithelial cells (ECs), blue arrow in (b) shows the side of the bronchium, and the red arrow shows the side of the capillary at the air-blood barrier having a continuous thin basement membrane. (c) Macrophages within the blood vessel. Arrow in (d) shows alveolar epithelial cells (AECs) with clear lamellar bodies. (e–h) Electron micrographs in the vehicle group demonstrated disruption of the basement membrane in capillaries (e); thickened air-blood barrier (f); perivascular edema and inflammatory cell infiltration (g); and an apoptotic AEC with visible empty lamellar bodies, swollen cell organs, and contracted nuclei with condensed and fragmented nuclear chromatin (arrow) (h). (i–l) Ultrastructural morphology of EC (i), thickened air-blood barrier (j), perivascular edema (k), and apoptotic AEC (l) in the TSP-1 overexpression group, which was similar to the vehicle group. (m–p) Depiction of normal ultrastructural morphology of EC (m), AEC (p), air-blood barrier (n), and alleviated perivascular edema (o) in the TSP-2 overexpression group. The scale bars in (a, c, e, f, i, j, l, and n) are 1 *μ*m; the scale bars in (b, d, g, h, k, m, o, and p) are 2 *μ*m (*n* = 5 per group).

Additional ultrastructural morphology analysis of the pulmonary tissue sections from normal mice (no treatment) using EM revealed normal blood vessel epithelial cells (Figure 8(a)), air-blood barrier with a thin basement membrane (Figure 8(b)), macrophages in the blood vessels (Figure 8(c)), and alveolar epithelial cells (AECs) with clear lamellar bodies (Figure 8(d)). However, lung sections from LPS-treated mice showed disruption of the basement membrane in the capillaries (Figure 8(e)); thickened air-blood barrier (Figure 8(f)); perivascular edema and inflammatory cell infiltration (Figure 8(g)); and apoptotic AECs with visible empty lamellar bodies, swollen cell organs, and contracted nuclei with condensed and fragmented nuclear chromatin (Figure 8(h)). A similar trend was observed in lung sections from TSP-1-overexpressing mice (Figures 8(i)–8(l)). However, lung sections from TSP-2-overexpressing mice after LPS treatment displayed relatively normal ultrastructural morphology of blood vessel epithelial cells (Figure 8(m)), air-blood barrier (Figure 8(n)), and alveolar epithelial cells (Figure 8(p)). In addition, we observed attenuated perivascular edema in these sections (Figure 8(o)).

### 3.6. TSP-2 Overexpression Diminishes Pulmonary Apoptosis in ARDS Mice

Since TSP-2 suppressed LPS-induced apoptosis *in vitro*, we next analyzed its effects *in vivo* by measuring caspase-3 expression using Western blot analysis and immunofluorescence staining of lung tissue sections from the four groups. Our results revealed that TSP-2 overexpression suppressed expression of active caspase-3 in pulmonary tissue compared to LPS-treated mice expressing empty vector or TSP-1 overexpression (Figures 6(c) and 6(d)). Immunofluorescence staining analysis further illustrated that lung tissues of LPS-treated mice with empty vector or TSP-1 overexpression had more caspase-3-positive cells (Supplementary Fig 3A, B, D, E, G, H, J, K, and N), while lung sections from TSP-2-overexpressing mice and normal control mice exhibited diffuse caspase-3 staining (Supplementary Fig 3C, F, I, L, M, and O).

### 3.7. TSP-2 Overexpression Induces M2 Macrophage Phenotype through Activation of PI3K Signaling *In Vivo*

**Figure 9:**
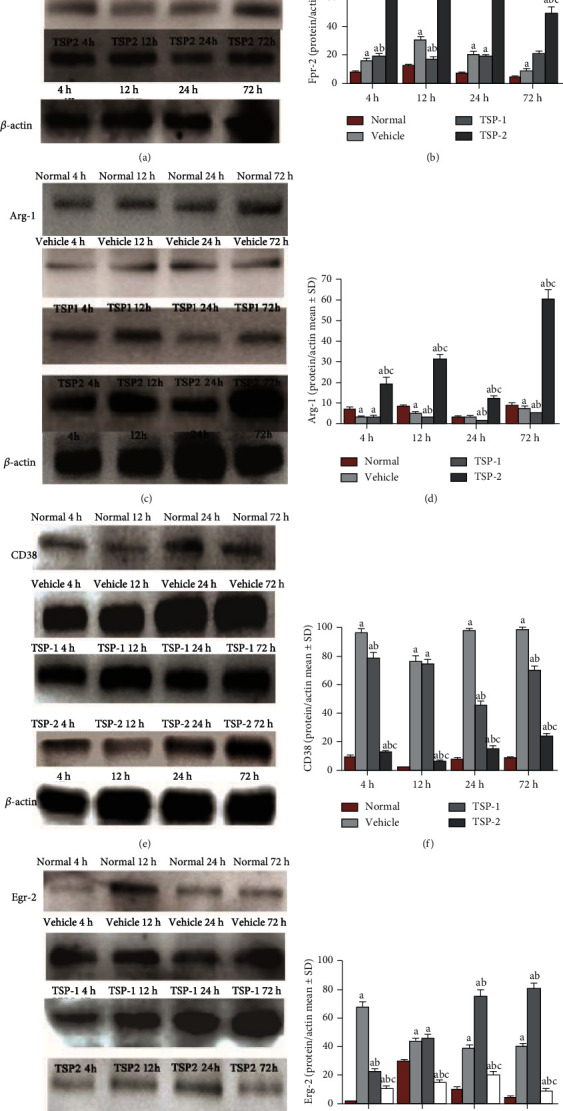
Effect of TSP overexpression on macrophage polarization *in vivo*. Mouse protein lysates from normal mice or mice treated with LPS for 4, 12, 24, and 72 h and overexpressing either empty vector (vehicle group), TSP-1 (TSP-1 group), or TSP-2 (TSP-2 group) were analyzed for expression of M2 (Agr-1 and Egr-2) (a, c) and M1 (CD38 and Fpr-2) (e, g) macrophage markers using Western blot analysis. *β*-Actin expression served as the loading control. The bar graphs in (b, d, f, and h) show the expression of these markers normalized to *β*-actin. The percentage of detected protein/actin is shown as mean ± SD. *P* value of < 0.05 represents significant difference. ^a^*P* < 0.05 compared with normal control; ^b^*P* < 0.05 compared with vehicle group; ^c^*P* < 0.05 compared with TSP-1 transfected group (*n* = 4 per group).

**Figure 10:**
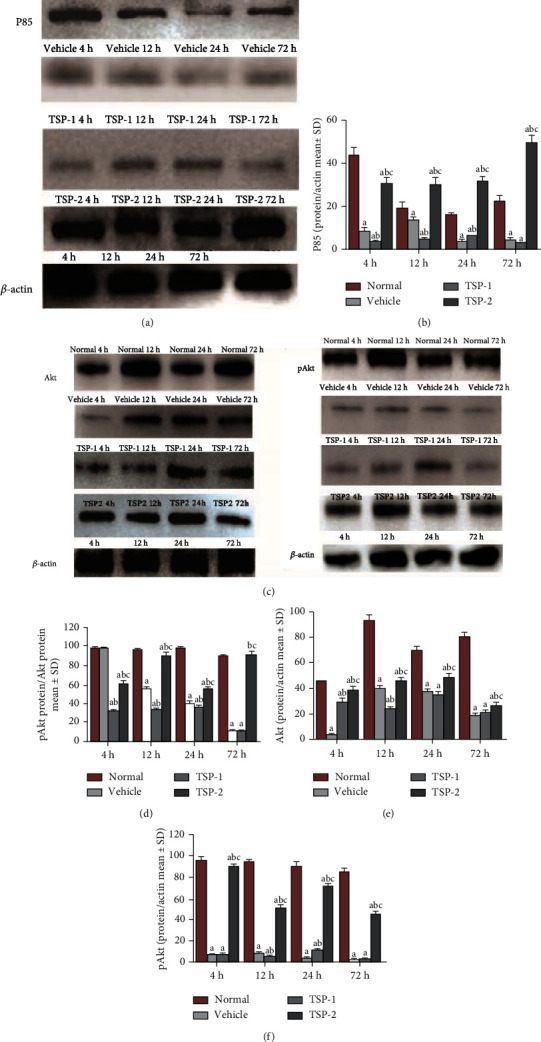
Effect of TSP overexpression on PI3K signaling *in vivo*. Mouse protein lysates from normal mice or mice treated with LPS for 4, 12, 24, and 72 h and overexpressing either empty vector (vehicle group), TSP-1 (TSP-1 group), or TSP-2 (TSP-2 group) were analyzed for p85 (a), p-Akt, and total Akt (c) expression using Western blot analysis. *β*-Actin expression served as the loading control. (b, e, and f) Bar graphs are shown after normalizing the expression of p85, total Akt, and p-Akt to *β*-actin. (d) Bar graph for p-Akt expression is shown after normalizing to total Akt expression. The percentage of detected protein/actin is shown as mean ± SD. *P* value of < 0.05 represents significant difference. ^a^*P* < 0.05 compared with normal control; ^b^*P* < 0.05 compared with vehicle group; ^c^*P* < 0.05 compared with the TSP-1 transfected group (*n* = 4 per group).

**Figure 11:**
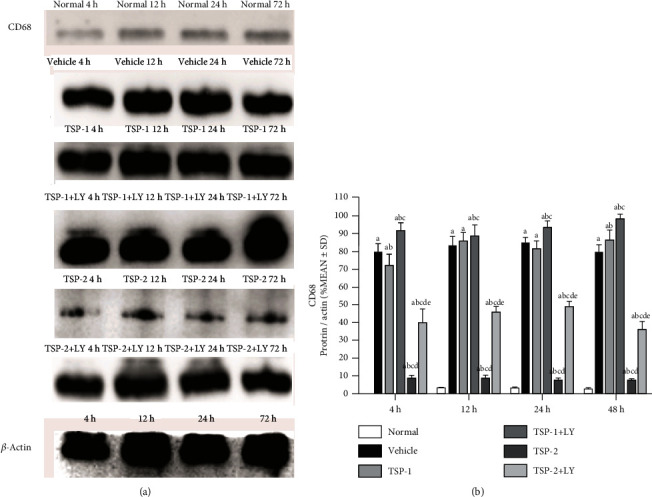
Effect of PI3K/Akt signaling inhibitor LY294002 on lung inflammation *in vivo*. The expression of CD68, a macrophage marker, in protein lysates of mice treated with LPS for 4, 12, 24, and 72 h and overexpressing either empty vector (vehicle group), TSP-1 (TSP-1 group), or TSP-2 (TSP-2 group), as well as the TSP-1/TSP-2+LY204002 groups, are shown using Western blot analysis. *β*-Actin expression served as the loading control. The percentage of detected protein/actin is shown as mean ± SD. *P* value of < 0.05 represents significant difference. ^a^*P* < 0.05 compared with normal control; ^b^*P* < 0.05 compared with vehicle control; ^c^*P* < 0.05 compared with the TSP-1 group; ^d^*P* < 0.05 compared with the TSP-1 transfected+LY294200 group; ^e^*P* < 0.05 compared with the TSP-2 transfected group (*n* = 4 per group).

We next analyzed the effect of TSP proteins on macrophage polarization and PI3K signaling in ARDS mice. Protein analysis of lung tissue lysates from mice in the four groups showed that expression of M2 phenotype markers, Egr-2 (Figures 9(a) and 9(b)) and Arg-1 (Figures 9(c) and 9(d)), increased significantly in the TSP-2-overexpressing mice at all time points after LPS treatment, while expression of M1 phenotype-specific markers, CD38 (Figures 9(e) and 9(f)) and Fpr-2 (Figures 9(g) and 9(h)), increased in TSP-1-overexpressing mice. *In vivo* analysis of the effect of TSP proteins on PI3K signaling showed a similar pattern to the *in vitro* analysis. TSP-1-overexpressing and vehicle lung tissue lysates showed reduced p85, p-Akt, and total Akt protein levels compared to normal control (Figures 10(a)–10(f)). However, TSP-2-overexpressing lung tissue lysates showed no significant change, and their protein levels were similar to control lysates, which suggested that TSP-2 overexpression reversed the negative effects of LPS on this signaling pathway. *In vivo* inhibition of the PI3K/Akt pathway using LY294002 resulted in a significant increase in CD68 expression in the TSP-2-transfected mice compared to the control mice. Additionally, these mice showed no remarkable differences in CD68 expression compared to the mice in the vehicle and TSP-1-transfected groups. CD68 expression was exacerbated in the TSP-1-transfected mice treated with LY294002 (Figure 11).

## 4. Discussion

In this study, we sought to delineate the role of TSP proteins in LPS-induced ARDS. Among the two TSP proteins that were analyzed, TSP-2 overexpression specifically and effectively attenuated LPS-induced ARDS by inhibiting apoptosis, pulmonary vascular permeability, edema, inflammation, and lung injury through inducing the PI3K signaling pathway. Furthermore, we observed that TSP-2 overexpression favored M2 macrophage polarization both *in vitro* and *in vivo*. In contrast, TSP-1 overexpression was not able to reverse the LPS-mediated effects in either the *in vitro* or *in vivo* model, and TSP-1 favored M1 macrophage polarization.

### 4.1. TSP-1

TSP-1 is a matricellular protein that has been described as an important factor in maintaining vascular structure and homeostasis through regulating various biological functions, such as cell proliferation, apoptosis, and adhesion [16]. Some studies have indicated that TSP-1 can promote monocyte migration to the injury site [17], where it typically promotes monocytic cell adhesion to extracellular matrix (ECM). Thus, TSP-1 expression plays a significant role in mononuclear cell migration and adhesion, and subsequently contributes to vascular inflammation. It has also been reported that macrophages derived from TSP-1-deficient mice had a reduced inflammatory phenotype, thereby indicating the role of TSP-1 in macrophage activation [18]. In the same study, it was demonstrated that human TSP-1 treatment stimulated TNF-*α* expression in bone marrow-derived macrophages in a time- and dose-dependent manner, and TSP-1-stimulated macrophage activation was TLR4 dependent [18]. Another study demonstrated that TSP-1 can act as a positive modulator of M1 differentiation in human monocytic cells [19]. Our current data, showing that TSP-1 overexpression induced the M1 phenotype in MH-S cells *in vitro* and ARDS *in vivo*, is consistent with these earlier publications.

### 4.2. TSP-2

The matricellular glycoprotein TSP-2 has been shown to regulate cell adhesion, migration, and proliferation. TSP-2 is also a strong inhibitor of angiogenesis [20]. In addition, inflammation associated with tissue injury has been identified as a powerful inducer of TSP-2 expression in murine tissues. TSP-2 has been shown to suppress the production of proinflammatory mediators, such as IFN-*γ* and TNF-*α*, and induce the depletion of tissue-residing T-cells [9]. Previous studies have also revealed that TSP-2 deficiency leads to an accelerated and increased influx of T-cells and monocytes/macrophages [20], and TSP-2-null mice display high inflammation, ECM deposition, and leakage of the blood-brain barrier (BBB) [21]. Consistently, we observed that TSP-2 overexpression in MH-S cells promoted the anti-inflammatory M2 phenotype, which was accompanied by increased expression of the anti-inflammatory cytokine IL-10 and M2 markers Arg-1 and Fpr-3. ARDS is characterized by an excessive inflammatory response within the lungs and severely impaired gas exchange, resulting from alveolar-capillary barrier disruption and pulmonary edema [22]. One of the most important hallmarks of ARDS is high infiltration and accumulation of leukocytes in both interstitial and alveolar spaces. LPS-induced ARDS typically exhibits visible endothelial-epithelial damage and capillary-alveolar permeability, along with lung edema and tissue damage [22]. During our analysis of lung morphology and pathology of TSP-2-overexpressing ARDS mice, we observed that TSP-2 resulted in anti-inflammatory features, characterized by a reversal of LPS-induced endothelial-epithelial damage, reduced capillary-alveolar permeability, and inhibition of neutrophil migration and infiltration. These observations indicate the potential mechanism underlying improved endothelial cell proliferation and inhibition of adhesion/transmigration of inflammatory cells on blood vessel endothelial cells [20].

LPS treatment induces inflammatory factors and recruits neutrophils into the intravascular space across the endothelium and epithelium, as well as into the lungs and alveolar space, thereby promoting tissue damage [23]. The polarization of macrophages into the M1 phenotype has been linked to lung tissue damage in ARDS. Our results demonstrated that TSP-1 overexpression promoted M1 macrophage polarization, which contributed to the deteriorating histopathology similar to LPS treatment of empty vector mice. Another independent study clearly demonstrated that M2 polarization significantly reduced lung inflammation and injury, reducing neutrophil influx into the lungs and augmenting apoptosis [24]. Consistent with this observation, TSP-2 overexpression in our study favored M2 macrophage polarization, and thus improved histopathologic changes in the lung tissue of TSP-2-overexpressing mice, as evidenced by decreases in lung injury and inflammation scores.

### 4.3. PI3K Signaling

TSP-2 is associated with the PI3K/Akt signaling pathway [25]. A previous study demonstrated that Akt, p-Akt, and PI3K expression were drastically reduced following TSP-2 silencing. Moreover, these cells exhibited enhanced apoptosis and reduced proliferation [25]. In human pulmonary microvascular endothelial cells, it has been observed that LPS-mediated dysregulation of barrier function involved PI3K/Akt signaling in a dose-dependent manner [26]. In addition, levels of p-Akt were dramatically reduced in the ARDS mouse model, and treatment with the PI3K inhibitor, wortmannin, further enhanced lung injury [27]. We observed consistent changes in PI3K/Akt signaling both after TSP-2 overexpression in MH-S cells *in vitro* and in the macrophage *in vivo* model. Inhibition of the PI3K/Akt pathway by LY294002 resulted in a significant increase in CD68 expression in mice transfected with TSP-2, as well as further exacerbated CD68 expression in mice transfected with TSP-1, thereby confirming the relationship between PI3K/Akt pathway activation and inflammation. This suggests that the effects of TSP-2 on suppressing inflammation were at least partly achieved through activation of the PI3K/Akt pathway. Consistent with our data, previous studies have also reported that genipin pretreatment significantly activated PI3K/Akt signaling and alleviated LPS-induced inflammation. However, LY294002 specifically inhibited the protective effects of genipin, including the effects on apoptosis, endoplasmic reticulum stress (ERS), and inflammation in an LPS-induced acute lung injury (ALI) model [28]. Another study showed that fibroblast growth factor-2 (FGF-2) effectively reduced LPS-induced inflammation, oxidative stress, and apoptosis in alveolar epithelial cells and increased activation of the PI3K/Akt signaling pathway. LY294002 treatment also alleviated the protective effect of FGF-2 in the lung tissue [29]. Therefore, it is reasonable to assume that activating the PI3K/Akt signaling pathway is beneficial for protecting the lung tissue. In addition, we showed that TSP-2 overexpression upregulated p-Akt, Akt, and p85 (a regulatory subunit of PI3K) following LPS treatment, and thus enhanced cell viability and inhibited apoptosis, which was consistent with previously published studies [30, 31].

However, we observed an opposite effect of TSP-1 on PI3K signaling in our *in vitro* and *in vivo* assays. Our *in vitro* study showed that TSP-1 overexpression restored p85 and Akt levels within 0.5 h of LPS treatment, while our *in vivo* data indicated that TSP-1 overexpression reduced p85, p-Akt, and total Akt protein levels compared to the normal control after 4, 12, 24, and 72 h of LPS injection. These differences can be attributed to the different time points at which expression levels were assessed. The early time point effects of TSP-1 overexpression on PI3K signaling can be due to its effect on severe pulmonary vascular remodeling, peripheral vascular rarefaction, and fibrosis, which can subsequently limit lung function at later stages. Consistent with this data, previous studies have also shown that *Akt1*^−*/*−^ mice were protected from chronic hypoxia-induced pulmonary vascular tissue remodeling and fibrosis, and TSP-1 can work as a matricellular protein in hypoxia-induced pulmonary remodeling [32]. In the same study, lungs from *TSP1*^−*/*−^ mice were also analyzed to determine resistance to pulmonary fibrosis induced by adMyrAkt1 overexpression.

### 4.4. TSP Overexpression as Gene Therapy

The potential of using TSPs as gene therapy has been explored for quite some time. In 2002, one study was the first to suggest that TSP-2 gene therapy can act as an antiangiogenic tumor therapy. Herein, a tissue-engineered implant system was created with an ability to continuously produce TSP-2 *in vivo*, with the intent to potently inhibit endogenous tumor growth and angiogenesis. In this system, fibroblasts were retrovirally transduced to overexpress TSP-2 and were seeded onto biodegradable polymer scaffolds. This bio-implant-generated TSP-2 potently inhibited tumor growth and angiogenesis of human squamous cell carcinomas, malignant melanomas, and Lewis lung carcinomas [33]. Similarly, other studies have also demonstrated the potential of TSP-2 gene therapy to ameliorate experimental glomerulonephritis *via* inhibiting cell proliferation, inflammation, and TGF-*β* activation. In one such study, TSP-2 overexpression inhibited both glomerular endothelial and mesangial cell proliferation, resulting in reduced glomerular cell number and glomerular tuft area [34]. Importantly, the inflammatory response, as monitored by T-cell and antigen-presenting cell infiltration, was also significantly reduced after TSP-2 overexpression [34]. In chronic allograft nephropathy, long-term TSP-2 gene therapy led to the inhibition of TGF-*β* activation, inflammation, and angiogenesis [35]. Together, these studies provide a strong foundation for translating TSP gene therapy to the clinic.

## 5. Conclusions

In conclusion, macrophages, which can be either inflammatory or anti-inflammatory, can overexpress various thrombospondins that can induce macrophage polarization. Subsequently, these polarized macrophages play different roles in pulmonary inflammation. Our study specifically demonstrated that TSP-1 overexpression promoted M1 macrophage polarization and enhanced LPS-induced inflammation, while TSP-2 overexpression effectively attenuated LPS-induced ARDS by promoting M2 macrophage polarization. These findings lead us to conclude that TSP-2 could be developed as a potential therapeutic target against LPS-induced ARDS.

## Data Availability

The datasets generated and analyzed during the current study are available from the corresponding authors on reasonable request.

## Supplementary Materials

Supplementary 1 Figure SP1 Expression analyses of TSP-1 and TSP-2 overexpression *in vivo*. The mouse tissue sections from normal mice or mice treated with LPS for 4, 12, 24, and 72 h and overexpressing either empty vector (vehicle group), TSP-1 (TSP-1 group), or TSP-2 (TSP-2 group) were analyzed for TSP-1 (A) and TSP-2 (C) protein expression using Western blot analysis. *β*-Actin expression served as a loading control. (B, D) represented TSP-1 and TSP-2 expressions after normalization to *β*-actin expression in all four groups. The expression percentage of detected proteins/actin is shown as mean ± SD. *P* value of < 0.05 represents significant difference. ^a^*P* < 0.05 compared with normal control; ^b^*P* < 0.05 compared with vehicle group; ^c^*P* < 0.05 compared with TSP-1 transfected group (*n* = 4 per group).

Supplementary 2 Figure SP2. TSP-2 effects on inflammatory cell accumulation in BALF. (A–M) Analyses of inflammatory cells using Wright-Giemsa staining in BALF collected from mice treated with LPS for 4, 12, 24, and 72 h and overexpressing either empty vector (vehicle group), TSP-1 (TSP-1 group), or TSP-2 (TSP-2 group). (M) Wright-Giemsa staining in BALF from normal mice. Scale bar = 100 *μ*m. (N) Five sections were randomly selected, and images were photographed under 400x magnification. Leukocyte numbers were counted from three fields per section. The number of leukocytes in each group is shown as mean ± SD. *P* value of < 0.05 represents significant difference; ^∗^*P* < 0.05*vs.* normal group; ^#^*P* < 0.05*vs.* vehicle group (*n* = 3 per group).

Supplementary 3 Figure SP3. Effect of TSP-2 on active caspase-3 *in vivo*. (A–L) Caspase-3 staining (red color) in lung tissue sections of mice treated with LPS for 4, 12, 24, and 72 h and overexpressing either empty vector (vehicle group), TSP-1 (TSP-1 group), or TSP-2 (TSP-2 group). (M) Caspase-3 staining in lung sections from normal mice. Scale bar = 100 *μ*m. (*n* = 5 per group).
