# Supplementary figures and images for: Contribution of Thrombospondin-1 and -2 to Lipopolysaccharide-Induced Acute Respiratory Distress Syndrome
Source: Mediators Inflamm. 2021 Apr 16;2021:8876484. doi: 10.1155/2021/8876484 (PMC8087994; doi:10.1155/2021/8876484)

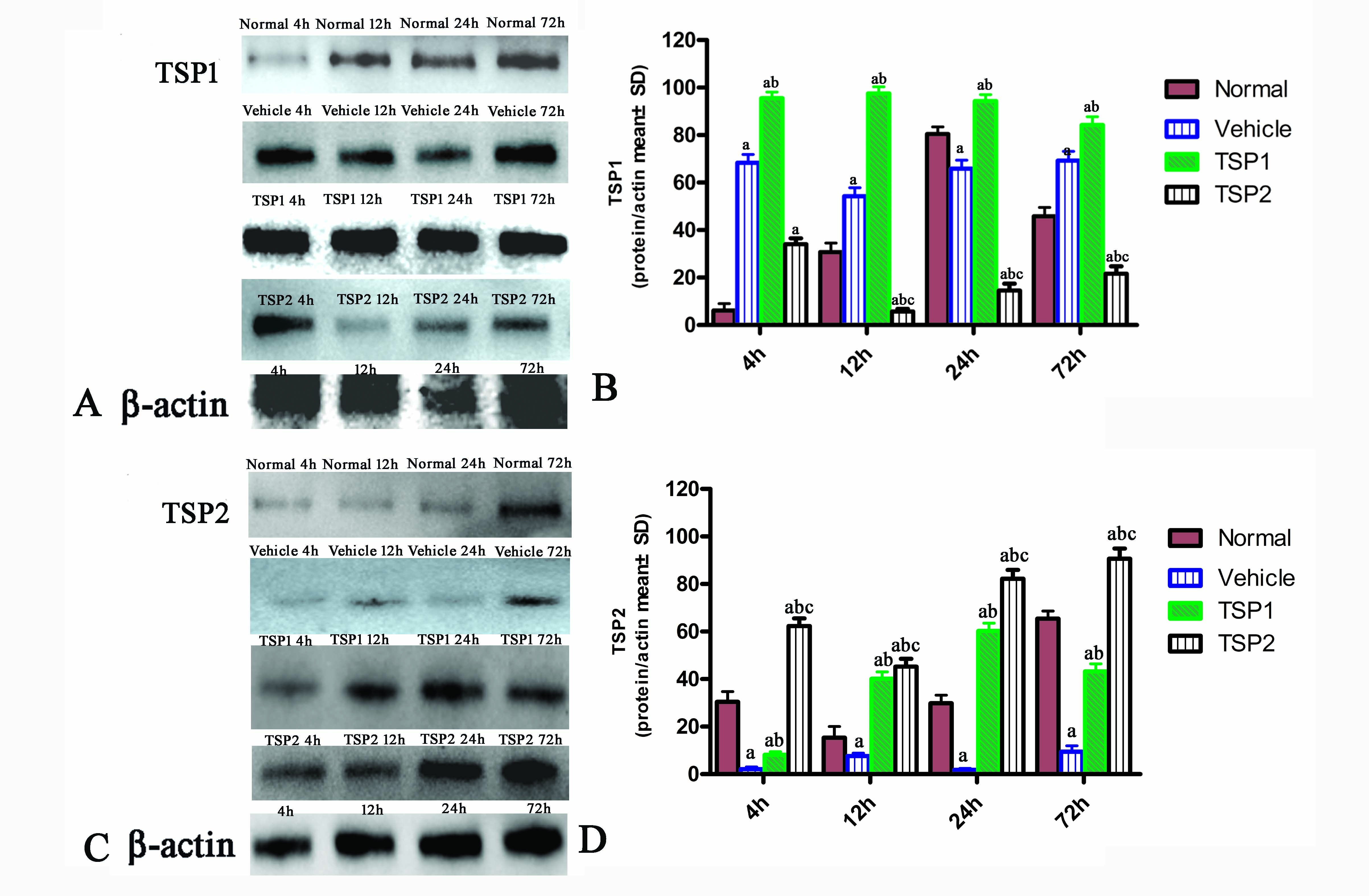

Supplement: Supplementary 1 — Figure SP1 Expression analyses of TSP-1 and TSP-2 overexpression in vivo. The mouse tissue sections from normal mice or mice treated with LPS for 4, 12, 24, and 72 h and overexpressing either empty vector (vehicle group), TSP-1 (TSP-1 group), or TSP-2 (TSP-2 group) were analyzed for TSP-1 (A) and TSP-2 (C) protein expression using Western blot analysis. β-Actin expression served as a loading control. (B, D) represented TSP-1 and TSP-2 expressions after normalization to β-actin expression in all four groups. The expression percentage of detected proteins/actin is shown as mean ± SD. P value of < 0.05 represents significant difference. aP < 0.05 compared with normal control; bP < 0.05 compared with vehicle group; cP < 0.05 compared with TSP-1 transfected group (n = 4 per group). [file 8876484.f1.jpg]

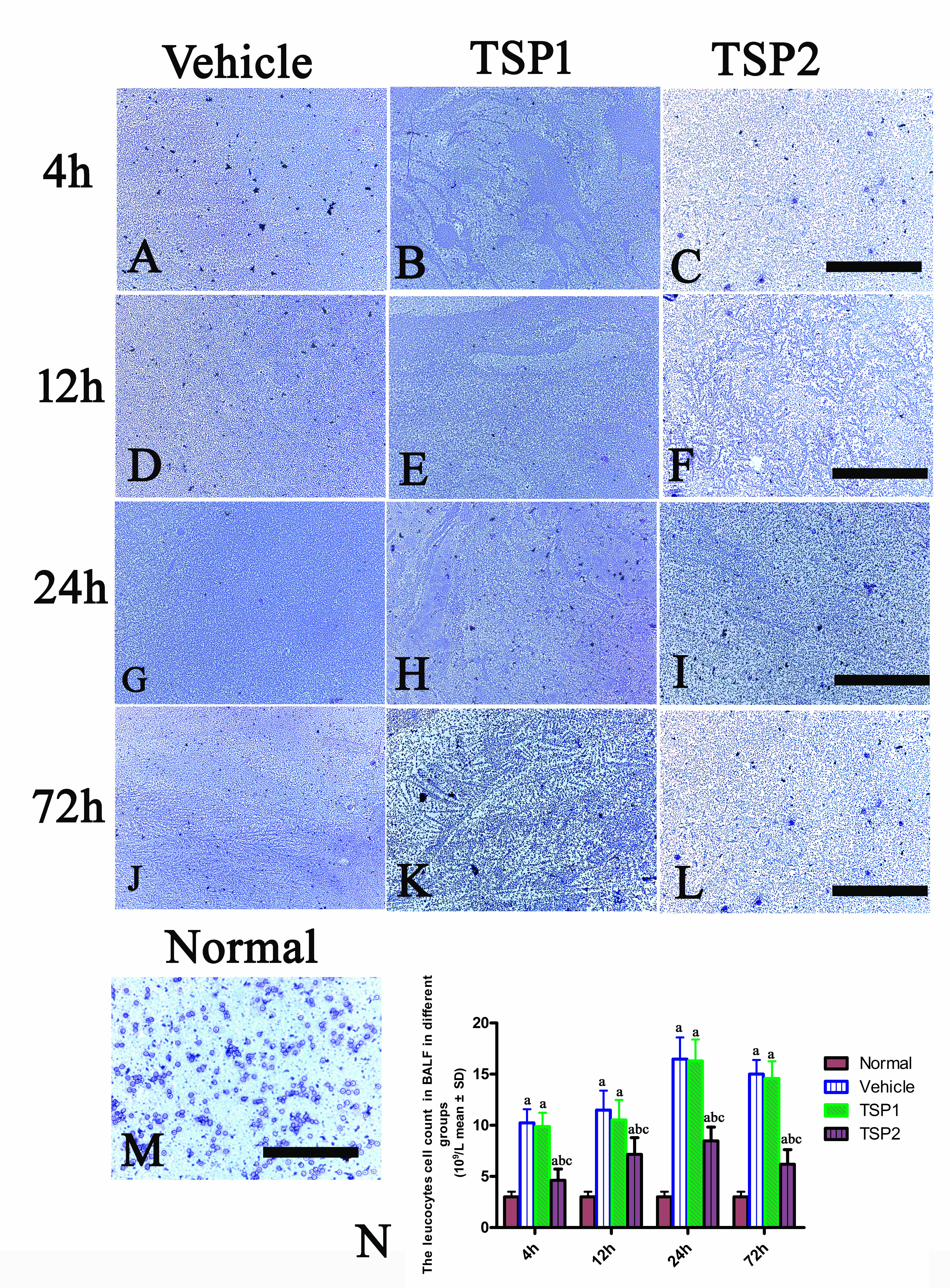

Supplement: Supplementary 2 — Figure SP2. TSP-2 effects on inflammatory cell accumulation in BALF. (A–M) Analyses of inflammatory cells using Wright-Giemsa staining in BALF collected from mice treated with LPS for 4, 12, 24, and 72 h and overexpressing either empty vector (vehicle group), TSP-1 (TSP-1 group), or TSP-2 (TSP-2 group). (M) Wright-Giemsa staining in BALF from normal mice. Scale bar = 100 μm. (N) Five sections were randomly selected, and images were photographed under 400x magnification. Leukocyte numbers were counted from three fields per section. The number of leukocytes in each group is shown as mean ± SD. P value of < 0.05 represents significant difference; ∗P < 0.05vs. normal group; #P < 0.05vs. vehicle group (n = 3 per group). [file 8876484.f2.jpg]

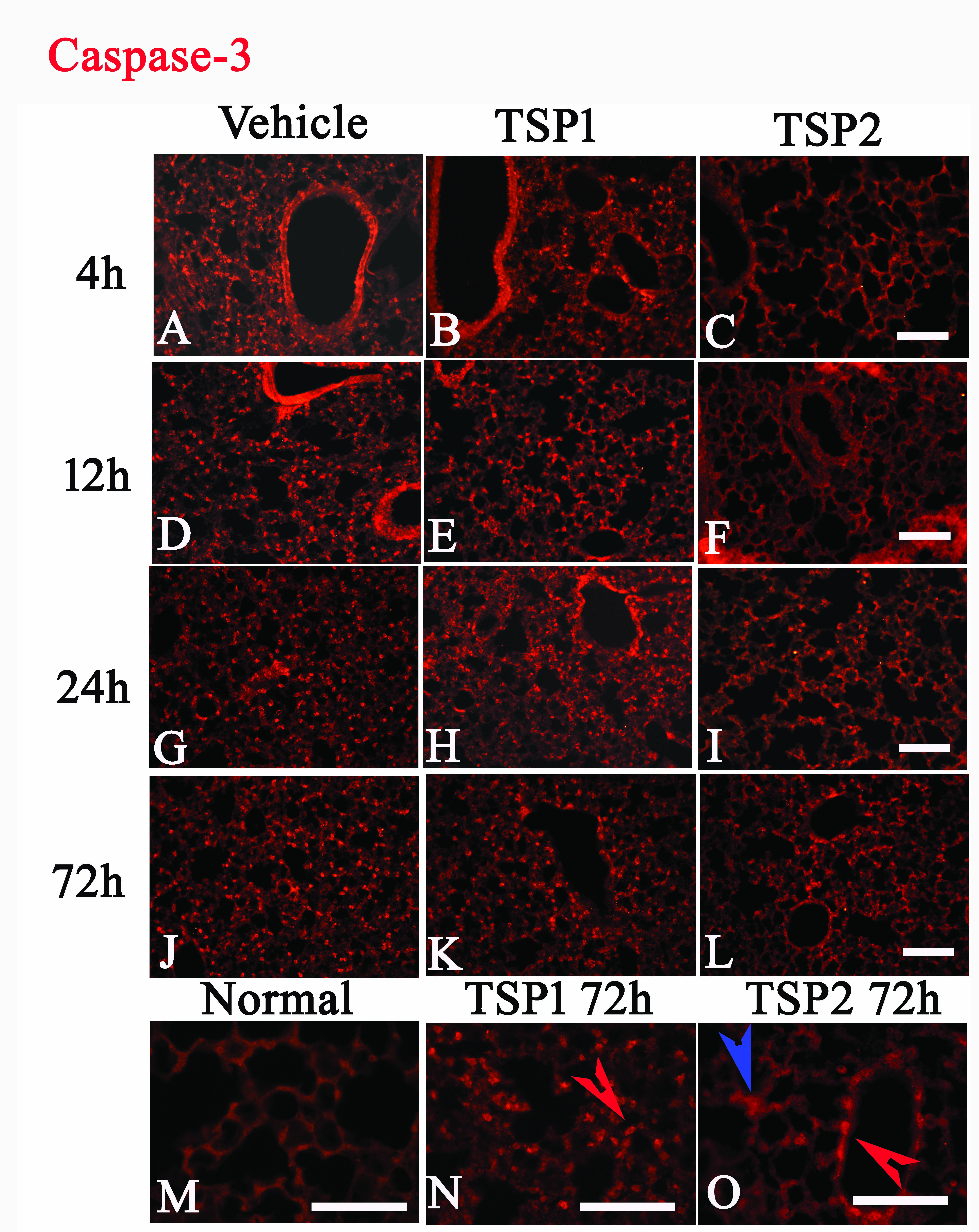

Supplement: Supplementary 3 — Figure SP3. Effect of TSP-2 on active caspase-3 in vivo. (A–L) Caspase-3 staining (red color) in lung tissue sections of mice treated with LPS for 4, 12, 24, and 72 h and overexpressing either empty vector (vehicle group), TSP-1 (TSP-1 group), or TSP-2 (TSP-2 group). (M) Caspase-3 staining in lung sections from normal mice. Scale bar = 100 μm. (n = 5 per group). [file 8876484.f3.jpg]
